# Dealing with an uncertain future: a survey study on what patients with chronic kidney disease actually want to know

**DOI:** 10.1093/ckj/sfae225

**Published:** 2024-07-19

**Authors:** Jet Milders, Chava L Ramspek, Yvette Meuleman, Willem Jan W Bos, Wieneke M Michels, Wanda S Konijn, Friedo W Dekker, Merel van Diepen

**Affiliations:** Department of Clinical Epidemiology, Leiden University Medical Center, Leiden, The Netherlands; Parnassia Groep, The Hague, The Netherlands; Department of Clinical Epidemiology, Leiden University Medical Center, Leiden, The Netherlands; Department of Internal Medicine, Leiden University Medical Center, Leiden, The Netherlands; Department of Internal Medicine, St. Antonius Hospital, Nieuwegein, The Netherlands; Department of Internal Medicine, Leiden University Medical Center, Leiden, The Netherlands; Dutch Kidney Patients Association, Bussum, The Netherlands; Department of Clinical Epidemiology, Leiden University Medical Center, Leiden, The Netherlands; Department of Clinical Epidemiology, Leiden University Medical Center, Leiden, The Netherlands

**Keywords:** CKD, dialysis, epidemiology, kidney transplantation, prognosis

## Abstract

**Background:**

Prognostic uncertainty is a recurring theme among patients with chronic kidney disease (CKD). We developed a survey to explore whether CKD patients want to know more about their future, and if so, which topics they prioritize. In addition, we explored differences between several subgroups.

**Methods:**

A survey was constructed and tested in collaboration with the Dutch Kidney Patients Association. The survey consisted of three parts: (i) demographics, (ii) considerations about the future, and (iii) prognostic information. The survey was distributed among CKD patients (all stages) through patient associations and via healthcare professionals in two Dutch hospitals. Descriptive statistics were used to summarize the results. All results were stratified by population, sex, and age.

**Results:**

A total of 163 patients (45 CKD, 26 dialysis, and 92 kidney transplantation) participated in the survey. The mean age was 63.9 (SD 12.0) and 48.5% was male. Most patients think about their future with CKD occasionally (56.4%) or often (35.0%). Nearly half of the patients (49.7%) discuss the future with their nephrologist, some (19.6%) do not but would like to, and 20 (15.3%) prefer not to. Most patients (73.6%) want more prognostic information, regardless of it being positive or negative. Key topics to receive prognostic information about were laboratory values, symptoms, and physical well-being. Dialysis patients prioritized mental over physical well-being. CKD patients without kidney replacement therapy (KRT) indicated thinking about, and discussing their future more regularly than KRT patients.

**Conclusions:**

Patients with CKD contemplate their future regularly and express interest in receiving prognostic information on a variety of topics. One in five patients currently do not discuss their future with CKD with their nephrologist, despite wanting to do so. These findings underline the need to tailor prognostic information provision to patients’ preferences, advocating more attention to this subject both in research and clinical practice.

KEY LEARNING POINTS **What was known:**

- Prognostic uncertainty is common among CKD patients, leading to feelings of fear and hopelessness.
- Previous research has indicated that patients desire more information about their future with CKD in general.

**This study adds:**

- Most CKD patients regularly think about their future and express an interest in receiving prognostic information. Notably, patients without KRT report thinking about the future with CKD more often than their KRT counterparts.
- Despite the desire for information, a significant portion of patients do not discuss their future with their nephrologist, highlighting a gap in communication.
- Patients prioritize several outcomes in terms of prognosis, including laboratory values, symptoms, and physical well-being, with different priorities observed between CKD stages (CKD, dialysis, and kidney transplantation).

**Potential impact:**

- Tailoring prognostic information provision to individual preferences can empower patients to better cope with CKD and make informed treatment decisions.
- Increased attention to patients' prognostic information needs can enhance patient-centred care and improve clinical outcomes.
- The findings underscore the importance of integrating patient-reported outcomes and preferences into clinical practice to better meet the needs of CKD patients.

## INTRODUCTION

Patients with chronic kidney disease (CKD) often have several comorbidities and symptoms, ranging from severe itch to fatigue. This affects many aspects of life, such as their ability to work, participate in social activities, and quality of life [1–3]. Additionally, patients are at increased risk of adverse outcomes, such as cardiovascular events, initiation of kidney replacement therapy (KRT; dialysis and kidney transplantation) and death [4, 5]. Thus, many patients grapple with feelings of fear and hopelessness on receiving this diagnosis [6]. Moreover, the disease is paired with uncertainty as the disease trajectory varies highly per individual. This struggle with the unknown can cause a variety of mental health issues such as depression and anxiety, and hinders patients in making plans for the future. Prognostic uncertainty is thus a recurring theme throughout the different CKD stages, and patients have expressed a wish for more information about their future [6–9]. Prognostic information provision, tailored to individual preferences and needs, can benefit patients in various ways. It can empower patients to plan and adapt to changes that come with having CKD, fostering an increased sense of control over their life. Furthermore, prognostic information is essential for informed shared decision making about the various complex treatment options, helping patients weigh the benefits and challenges associated with different treatment options.

Although prognostic uncertainty is commonly present in CKD patients, the extent to which patients want to receive information about their potential future may vary per individual. Where some may want to know as much as possible about what the future has in store for them, others may prefer not to know what awaits. This wish for more information may also depend on the topic and context; for example, whether something can be done to prevent a complication, or whether information may aid in making a treatment decision. Moreover, preferences for information provision may differ depending on patient characteristics such as gender, age, or CKD stage. Understanding such

differences can aid healthcare professionals in tailoring communication to the individual in front of them.

Although research exists on the experience of prognostic uncertainty among CKD patients, to our knowledge, little research has been performed to identify preferences in terms of prognostic information provision. By identifying the topics patients want more prognostic information on, more attention can be paid to these topics in both clinical practice and future research. For example, prognostic models can then be developed for a broader spectrum of outcomes—for clinical outcomes and patient-reported outcomes [10, 11]. Therefore, this study aims to explore: (i) whether CKD patients want to know more about their future; (ii) and if so, which topics they find most important regarding their prognosis; and (iii) differences between several subgroups [CKD stage (CKD without KRT, dialysis, and kidney transplantation), gender and age].

## MATERIALS AND METHODS

To ensure transparent reporting, the Checklist for Reporting of Survey Studies (CROSS) was adhered to (Supplemental Table S1) [12].

### Ethics

This study is not subject to the Medical Research Involving Human Subjects Act (WMO) and a non-WMO declaration was issued by the Division Scientific Committee of the department of Clinical Epidemiology at Leiden University Medical Center (LUMC) in the Netherlands [13].

### Survey development and testing

To gather information on what patients with CKD want to know about their future, a survey was constructed by an expert panel consisting of researchers (J.M., C.L.R., F.W.D., and M.v.D.) and nephrologists (W.J.W.B. and W.M.M.) experienced in the development of surveys, patient-reported outcomes (PROs) and prognostic research. Additionally, we used literature to identify important PROs for our survey. Finally, meetings were organized with patient representatives of the Dutch Kidney Patients Association (NVN) so that patient input could be incorporated during the development stage of the survey [14]. Castor Electronic Data Capture System was used to make a web-based version of the survey and a paper version was also constructed so that patients without online access were able to participate as well. The survey was tested during a two-phase pilot in collaboration with volunteers of the Dutch Kidney Patients Association (Supplementary Materials). The final survey consisted of three parts: (i) demographics, (ii) considerations about the future, and (iii) prognostic information. For details, see the full translated survey and accompanying informational letter in the Supplementary Materials. The open-ended questions will be analysed separately in a separate qualitative study. For most multiple-choice questions and at the end of the survey, participants were given the opportunity to add any additional comments so that no important topics would be missed. These data were used in addition to the data from the multiple-choice questions in this paper.

### Study population and recruitment

The survey was developed for patients diagnosed with CKD, including patients receiving KRT (dialysis or kidney transplantation). For the sample size calculation, we considered our main question of how many kidney patients have a wish for more information about their future. Based on the binomial distribution, the required sample size was calculated using the following equation:


\begin{equation*}
n = \frac{{{{z}^2} \times \hat{p}\left( {1 - \hat{p}} \right)}}{{{{ \varepsilon }^2}}}
\end{equation*}

where *n* is the required sample size, *z* is the *z*-value for the confidence interval we want to compute, p̂ the proportion we want to demonstrate and ε the margin of error we allow in our estimate of the studied proportion (or the width of the confidence interval). Thus, if we wanted to demonstrate a proportion of 20% with a margin of error of 10% with 95% confidence, that is, we wanted to obtain a 95%-CI of [10%;30%] for the proportion of patients with a wish for more information about their future, the required sample size is 62. Should the proportion be actually 50%, a sample size of 96 is required for a 95%-CI of [40%;60%]. We aimed to gather at least 150 completed surveys. The web-based survey was first deployed through the national Dutch Kidney Patients Association and the regional Kidney Patients Association Diavaria (Leiden and surroundings). To reach a diverse group representing the Dutch CKD population, the survey was also distributed among patients via nephrologists, nurse practitioners, and one researcher (J.M.) in two hospitals (LUMC and Sint Antonius Hospital Nieuwegein).

### Data collection and analysis

The survey was fully anonymous, meaning that answers could not be traced back to participants. All surveys were completed between 17 October 2022 and 13 March 2023, and gathered in Castor EDC. Records were screened for duplicates that were hereafter removed. Patients were able to leave the visual analogue scale questions and open-ended questions blank, potentially resulting in missing data. Available case analysis was performed for all questions. Descriptive statistics were used to summarize the results. All results were stratified by CKD population (CKD without KRT, dialysis, and kidney transplantation), gender (male and female), and age (≤65 and >65) to explore differences between subgroups. Furthermore, a sensitivity analysis was performed to evaluate whether results differed based on whether patients were recruited via the Dutch Kidney Patients Association, Diavaria, LUMC, or St. Antonius Hospital Nieuwegein. R version 4.2.2 (R Foundation for Statistical Computing, Vienna, Austria) was used to compute all analyses.

## RESULTS

### Response and participant characteristics

The survey was sent to 393 patients of the Dutch Kidney Patients Association, of whom 92 (23.4%) completed the survey and eight (2.0%) filled it in partially. Of the 137 patients that received the survey through Diavaria, 16 (11.7%) completed it and two (1.5%) partially finished it. At the LUMC, 16 additional surveys were completed, and at the St. Antonius hospital 28 and one survey(s) were filled in completely and partially, respectively. Six duplicates were removed. Finally, a total of 152 patients filled in the survey completely and 11 patients partially.

The general participant characteristics are summarized in Table 1. In short: the mean (SD) age was 63.9 (12.0) years and 48.5% were male. Of the 163 patients, 45 (27.6%) patients did not receive any type of KRT, 26 (16.0%) patients received dialysis treatment, and 92 (56.4%) patients had received a kidney transplantation. Of the 26 dialysis patients, two patients were on the waiting list for a kidney transplantation.

**Table 1:** General characteristics of participants.

	Total (*n* = 163)	CKD (*n* = 45)	Dialysis (*n* = 26)	KTx* (*n* = 92)
Source of patient recruitment				
Dutch Kidney Patients Association	100 (61.3%)	17 (37.8%)	8 (30.8%)	75 (81.5%)
Diavaria*	18 (11.0%)	3 (6.7%)	3 (11.5%)	12 (13.0%)
Leiden University Medical Center	16 (9.8%)	2 (4.4%)	14 (53.8%)	0 (0%)
Sint Antonius Hospital Nieuwegein	29 (17.8%)	23 (51.1%)	1 (3.8%)	5 (5.4%)
Age (mean, SD)	63.9 (12.0)	65.4 (12.9)	70.6 (11.5)	61.4 (11.0)
Gender (male, %)	79 (48.5%)	22 (48.9%)	10 (38.5%)	47 (51.1%)
Education level				
Low	41 (25.2%)	14 (31.1%)	10 (38.5%)	17 (18.5%)
Medium	36 (22.1%)	9 (20.0%)	5 (19.2%)	22 (23.9%)
High	83 (50.9%)	20 (44.4%)	10 (38.5%)	53 (57.6%)
Other	2 (1.2%)	2 (4.4%)	0 (0%)	0 (0%)
Living situation				
Alone	35 (21.5%)	12 (26.7%)	7 (26.9%)	16 (17.4%)
Together with a partner	114 (69.9%)	31 (68.9%)	15 (57.7%)	68 (73.9%)
Child(ren) living at home	27 (16.6%)	6 (13.3%)	2 (7.7%)	19 (20.7%)
Care facility	3 (1.8%)	0 (0%)	2 (7.7%)	1 (1.1%)
Other	4 (2.5%)	1 (2.2%)	0 (0%)	3 (3.3%)
Cause of kidney disease				
Diabetes mellitus	11 (6.7%)	5 (11.1%)	1 (3.8%)	5 (5.4%)
Vascular disease	15 (9.2%)	8 (17.8%)	3 (11.5%)	4 (4.3%)
Glomerulonephritis	16 (9.8%)	4 (8.9%)	0 (0%)	12 (13.0%)
Pyelonephritis, kidney damage by medication, or nephrolithiasis	9 (5.5%)	2 (4.4%)	2 (7.7%)	5 (5.4%)
Polycystic kidney disease	35 (21.5%)	8 (17.8%)	5 (19.2%)	22 (23.9%)
Autoimmune disease	16 (9.8%)	4 (8.9%)	1 (3.8%)	11 (12.0%)
Cancer	3 (1.8%)	2 (4.4%)	0 (0%)	1 (1.1%)
Unknown	31 (19.0%)	9 (20.0%)	4 (15.4%)	18 (19.6%)
Other	27 (16.6%)	3 (6.7%)	10 (38.5%)	14 (15.2%)
Self-reported kidney function (eGFR)				
>60 ml/min/1.73 m^2^	30 (18.4%)	3 (6.7%)	1 (3.8%)	26 (28.3%)
45–59 ml/min/1.73 m^2^	33 (20.2%)	5 (11.1%)	0 (0%)	28 (30.4%)
30–44 ml/min/1.73 m^2^	32 (19.6%)	9 (20.0%)	0 (0%)	23 (25.0%)
15–29 ml/min/1.73 m^2^	25 (15.3%)	19 (42.2%)	0 (0%)	6 (6.5%)
<15 ml/min/1.73 m^2^	30 (18.4%)	7 (15.6%)	22 (84.6%)	1 (1.1%)
Unknown	13 (8.0%)	2 (4.4%)	3 (11.5%)	8 (8.7%)
Time since CKD diagnosis				
0–4 years	17 (10.4%)	13 (28.9%)	3 (11.5%)	1 (1.1%)
5–10 years	25 (15.3%)	12 (26.7%)	6 (23.1%)	7 (7.6%)
>10 years	121 (74.2%)	20 (44.4%)	17 (65.4%)	84 (91.3%)
Dialysis modality		NA		NA
Haemodialysis in hospital			24 (92.3%)	
Haemodialysis at home			1 (3.8%)	
Peritoneal dialysis			1 (3.8%)	

*KTx = kidney transplantation, Diavaria = regional kidney patients association covering the city of Leiden and surroundings.

### Considerations about the future with CKD

**Figure 1:**
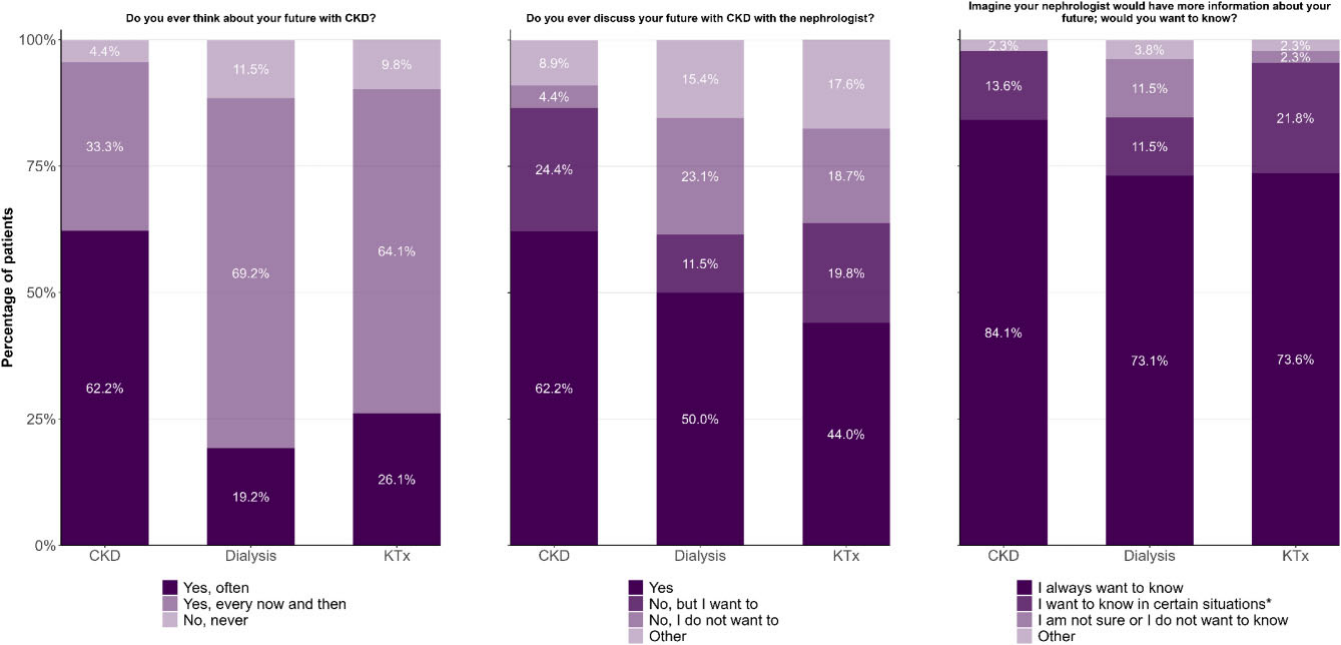
CKD = chronic kidney disease without KRT, KTx = kidney transplantation CKD (*n* = 45), dialysis (*n* = 26), KTx (*n* = 92). * ‘Certain situations’ consist of the following three response options: only in case of good news, if we can do something to prevent it, and if it supports me in making a treatment decision.

Most patients reported thinking about their future with CKD occasionally (56.4%) or often (35.0%). Patients not receiving KRT, reported thinking about their future often (62.2%) considerably more than those receiving dialysis (19.2%) or kidney transplantation (26.1%). The responses to this question, stratified by population, are shown in Fig. 1. After stratifying by gender and age (Supplementary Figures S1 and S2), we found that women more often report thinking about their future than men (73.9% versus 50.0%). Patients older than 65 years report thinking about their future more than younger patients.

Half of the patients (50.0%) reported that they discuss the future with their nephrologist and 35.2% answered that they do not. The remaining patients (14.8%) chose ‘Other’, of which most reported that it is currently not needed (e.g. due to a stable kidney function), but that this may change over time. Of those not discussing the future (*n* = 57), a substantial part (56.1%) reported wanting to do so. More CKD patients without KRT discuss their future with the nephrologist (62.2%) compared to dialysis (50.0%) and kidney transplantation patients (44.0%). However, more CKD patients without KRT do not discuss their future with the nephrologist, despite wanting to do so (24.4%) compared to dialysis (11.5%) and kidney transplantation patients (19.8%) (Fig. 1). Men report discussing their future with the nephrologist more often than women [63.6% vs. 60.9% (CKD without KRT); 60.0% vs. 40.0% (dialysis); and 52.1% vs. 35.5% (kidney transplantation)]. Additionally, more women than men currently do not discuss the future with their nephrologist despite wanting to do so [30.4% vs. 18.2% (CKD without KRT); 13.3% vs. 10.0% (dialysis); and 28.9% vs. 10.9% (kidney transplantation)] (Supplementary Figure S3). No clear differences based on age were detected (Supplemental Figure S4).

When asked: ‘Imagine your nephrologist would have more information about your future; would you want to know?’, most patients (76.4%) answered that they would always like to know, even if it is bad news. Some patients (20.4%) reported that they only want to know in certain situations, including when it concerns good news, when something can be done to prevent the outcome in question and/or when the information helps to make a treatment decision. Only a limited number of patients was unsure whether they would like to know (1.3%) or preferred not to know anything about their prognosis at all (1.9%). More CKD patients without KRT opted for the ‘I always want to know, even if it is bad news’ option (84.1%) than dialysis (73.1%) and kidney transplantation patients (73.6%) (Fig. 1). No clear differences based on gender or age were found (Supplemental Figures S5 and S6).

### Prognostic information

Participants rated their level of interest in obtaining additional prognostic information across nine topics on a scale of 0 to 100 (see Supplementary Materials). In short, the top three topics were: (i) laboratory values and measurements, (ii) symptoms, and (iii) physical well-being. Results across the three CKD populations were remarkably similar. Notably, only mental well-being was rated higher than physical well-being in dialysis patients. In Table 2, mean (SD) scores per topic, stratified by CKD population are presented. Mean scores were generally higher among CKD patients without KRT compared to dialysis and kidney transplantation patients, indicating a larger desire for prognostic information. Dialysis patients seem to be less interested in prognostic information surrounding a potential kidney transplantation [mean (SD): 42.3 (42.7)], and vice versa [mean (SD): 34.9 (33.3)]. Mean (SD) scores per topic, stratified by gender and age are presented in Supplementary Tables S2 and S3. No clear differences based on gender were detected. Younger patients reported higher mean scores across all nine topics. No additional differences based on age were found.

**Table 2:** Mean (SD) rating per topic.

	All (*n* = 163)	CKD* (*n* = 45)	Dialysis (*n* = 26)	KTx* (*n* = 92)
Laboratory values and measurements	77.8 (29.7)	80.6 (28.9)	74.4 (30.9)	77.3 (29.9)
Symptoms	68.0 (29.4)	73.0 (28.9)	64.2 (36.2)	66.6 (27.5)
Physical well-being	67.6 (34.3)	73.9 (30.7)	63.2 (36.4)	65.6 (35.4)
Mental well-being	61.4 (36.3)	68.2 (34.1)	63.5 (35.1)	57.3 (37.6)
Social participation	57.6 (37.1)	63.8 (34.7)	51.8 (39.8)	56.3 (37.5)
Disease progression and comorbidities	56.5 (34.1)	60.3 (33.9)	56.9 (38.5)	54.5 (33.1)
Kidney transplantation	52.5 (40.3)	61.3 (39.2)	42.3 (42.7)	51.1 (39.8)
Dialysis	47.0 (36.3)	62.3 (33.3)	62.8 (36.8)	34.9 (33.3)
Conservative management	38.4 (34.7)	48.8 (34.3)	22.6 (34.5)	37.9 (33.5)

**Figure 2:**
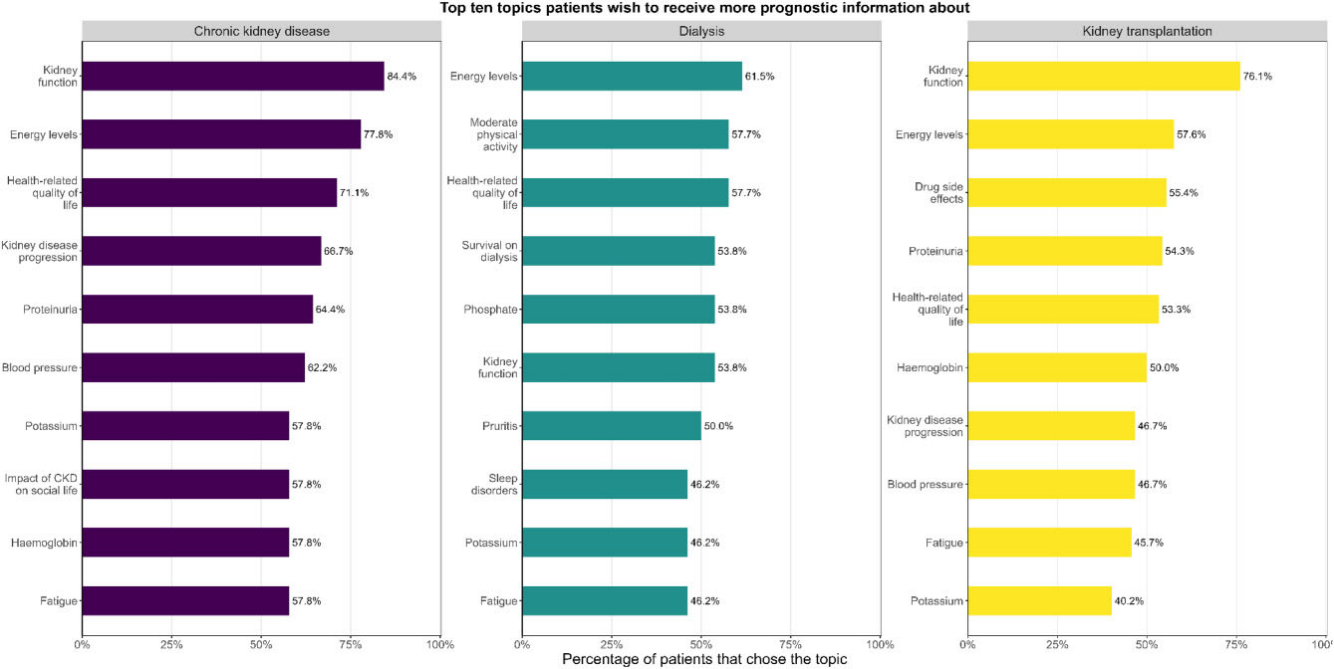
For dialysis patients, haemoglobin was also chosen by 46.2% of the patients. Moderate physical activity includes activities like climbing stairs, light household chores, walking, cycling, etc.

Patients were presented several specific outcomes per topic and were asked whether they would like to receive prognostic information about it (Fig. 2). Overall, outcomes that were chosen most often were kidney function, energy levels, and health-related quality of life (HRQOL). CKD patients without KRT rated the same three topics as most relevant. Dialysis patients most often chose energy levels, moderate physical activity and HRQOL, and the top three for kidney transplantation patients were kidney function, energy levels, and medication side effects. The top 10 most highly ranked outcomes had substantial overlap between the three CKD populations. However, there were also notable differences. For example, ‘impact on social life’ was unique to the top 10 of CKD patients without KRT. Outcomes that were only ranked in the top 10 by dialysis patients, included moderate physical activity, survival, phosphate, pruritis, and sleep problems. Finally, the only outcome that was unique to the top 10 for kidney transplantation patients was medication side effects. The top 10 chosen outcomes per population, stratified by gender and age are presented in Supplementary Figures S7–S12. Although the top 10 outcomes were similar when comparing men and women (albeit differently ranked), small differences were detected. For CKD without KRT, men focused on laboratory values and measurements, while women showed interest in survival and stress/anxiety. For dialysis patients, men showed interest in laboratory values and measurements (e.g. potassium and blood pressure), while women focused more on symptoms (e.g. sleep problems and muscle cramps). When stratifying for age, older patients prioritized outcomes such as concentration and memory problems, while younger patients focused more on physical symptoms such as restless legs and pain.

### Sensitivity analysis

No considerable differences were found after stratification by source (Dutch Kidney Patients Association, Diavaria, LUMC, or St. Antonius Hospital), see Supplementary Figure S13.

## DISCUSSION

Our survey study showed that most patients with CKD think about their future regularly. While many patients want to discuss this with their nephrologist, a considerable proportion does not do this. Most patients also want to know more about their prognosis, even if it is bad news. Finally, patients express interest in receiving more prognostic information for a variety of topics such as kidney function, energy levels, and quality of life.

Our survey is among the first to explore whether patients with CKD have a wish for more prognostic information, whether they want to discuss their future with CKD with the nephrologist, and which specific topics they consider important in terms of prognosis. However, the study comes with some limitations. First, patients were only recruited through Dutch patient associations and hospitals, and the questionnaire was only available in Dutch, making it difficult to generalize to immigrant patients, patients with different cultural backgrounds, and/or patients from other countries. Second, despite our efforts to include a wide variety of CKD patients, only a limited number of dialysis patients responded to our survey, hereby also limiting the generalizability. Additionally, we asked patients about their prioritized topic, but did not gather information on preferred timeframes for prognostic information. Short-term prognostic information is often the most actionable and relevant for clinical decision making. However, providing a longer-term outlook also holds significant value, as predicting outcomes over extended periods offers a broader perspective on what the future holds for this patient. Therefore, this needs to be evaluated further in future research. We also did not gather information on pre-emptive kidney transplantations. In future research, it would be interesting to assess whether results differ in these patients. Third, patients who are more inclined to complete surveys may represent a specific subgroup within the broader patient population. For example, individuals who prefer not to engage with healthcare professionals regarding prognostic matters might be less likely to participate in such surveys. Moreover, in our survey, >50% of patients had a high educational background. It is, therefore, unclear to which extent our subset of patients is representative of the entire Dutch CKD population. Finally, although surveys can efficiently gather a large amount of data from a broad sample of participants, they may not provide in-depth insights into a certain topic. In future research, this topic could be explored more thoroughly by conducting qualitative research (e.g. feelings that patients experience regarding their future with CKD).

In our study, CKD patients without KRT indicated thinking about their future more regularly than dialysis and kidney transplantation patients. Moreover, they reported discussing their future with the nephrologist more often than KRT patients. Although specific literature is lacking, these results could be explained by the fact that patients experience more prognostic uncertainty in the phases before starting KRT. They are newer to their diagnosis, are less experienced, and have a less clear disease and treatment trajectory ahead of them. The prospect of adapting to a life with CKD and the uncertainties surrounding disease progression may be particularly unsettling during this earlier phase [15]. Furthermore, patients in the phases before KRT are confronted with a variety of hypothetical scenarios: questions about KRT initiation, potential treatment side effects, and the overall impact on their quality of life become focal points of concern. The higher frequency of discussions about the future with nephrologists among those not yet on KRT, may stem from the urge to seek clarity. Patients in the early phases may engage in these conversations more as a means of gaining insights into the potential disease trajectories and the implications of various treatment options. In contrast, patients already on KRT, having traversed the initial decision-making phase, may experience less urgency for such discussions.

We found that women tend to think about their future with CKD more regularly than men. Despite thinking about the future less frequent, men report that they discuss it with their nephrologist more often. In line with our results, previous studies have shown that women often report worrying more than men, and that women are more prone to experience anxiety [16, 17]. Understanding potential differences between men and women can be important for tailoring support and communication strategies to the varying preferences of CKD patients. Literature on this topic, however, seems to be lacking. In our study, we did not find any notable differences based on age, which is in line with previous research on question-asking behaviour [18].

Remarkably, topics related to treatment decisions (dialysis, kidney transplantation and conservative management) were not prioritized by patients. A potential explanation may be that these topics are already extensively touched on by healthcare professionals in contrast to other outcomes that were prioritized more by the participants of our survey. In addition, it might be the case that these important treatment decisions, raise questions about the future for many patients during only a relatively short period. Once a decision has been made, the need for ongoing prognostic information in this area may reduce. Consequently, daily symptoms and quality-of-life issues might become a larger source of concern. Finally, from previous research we have learned that treatment-related outcomes are not always prioritized over PROs such as fatigue [19].

Although, to our knowledge, this is the first study specifically investigating the prognostic uncertainty among CKD patients in a quantitative manner, our results are in line with results found in previous, predominantly qualitative studies. First, patients reported that, despite experiencing feelings of fear, they want to know about their CKD diagnosis and its consequences early on, even if it would not influence clinical management of the disease [20]. Second, CKD patients have said to be interested in discussing predicted risks for several outcomes, including disease progression, mortality, and cardiovascular events. More specifically, patients mentioned that predictions regarding disease progression could aid them in planning their life, motivate them to better manage the disease, provide them with more timely information about the disease, and potentially comfort them [7, 21]. Third, in a recent qualitative study, most patients reported to be interested in receiving prognostic information, as it may motivate them to manage risk factors and it would allow them to better plan their life [22].

Currently, a discrepancy exists between the wish for prognostic information and the experienced information provision among CKD patients. Evidently, prognostic information is not always easily available. In previous research, patients have reported that discussing certain topics is very valuable, even when healthcare providers do not have all the answers or a solution. Patients explained it is important that they feel heard and understood by their doctor [23, 24]. Besides listening attentively, doctors have several options for giving patients more insight into their future. First, healthcare providers can discuss the expected disease trajectory of the patient based on their clinical expertise. For instance, in the stages preceding the initiation of KRT, it is common for healthcare providers to discuss prognostic information to facilitate informed treatment choices for their patients. To support this process and to better inform patients, there are several patient decision aid tools available, alongside dashboards displaying relevant disease-related data [25]. Additionally, a wide array of literature exists on the evolution of various outcomes in CKD patients. For example, studies exist on the changes in HRQOL and symptom burden before and after the start of dialysis [26, 27]. Although this information is not tailored to the individual patient, it can still be used to give a general expectation of the future with CKD. Finally, prognostic prediction models can be used as tools to support individualized prognostic information provision [11]. By predicting an individual's risk of a certain outcome, both the healthcare provider and the patient will have a better understanding of the likely disease trajectory of that individual. This information may help patients to feel more in control in coping with the disease and can help in making treatment decisions [7, 21].

## CONCLUSION

Most patients with CKD contemplate their future regularly and would like to receive individualized prognostic information on a variety of topics. Many patients currently do not discuss their future with CKD with their nephrologist, despite wanting to do so. These findings underline the need for tailored prognostic information provision to meet patients’ needs and for more attention to prognosis in future research and clinical practice.

## Supplementary Material

sfae225_Supplemental_File

## Data Availability

An Excel file with all data accompanied by a data dictionary can be shared on reasonable request to the corresponding author.
