# Supplementary material for: Dealing with an uncertain future: a survey study on what patients with chronic kidney disease actually want to know
Source: Clin Kidney J. 2024 Jul 19;17(8):sfae225. doi: 10.1093/ckj/sfae225 (PMC11345639; doi:10.1093/ckj/sfae225)
Supplement: sfae225_Supplemental_File [file sfae225_supplemental_file.docx]

SUPPLEMENTAL MATERIAL FOR

**Dealing with an uncertain future; A survey study on what patients with chronic kidney disease actually want to know**

Jet Milders^1^, Chava L. Ramspek^2^, Yvette Meuleman^1^, Willem Jan W. Bos^3,4^, Wieneke M. Michels^3^, Wanda S. Konijn^5^, Friedo W. Dekker^1^, Merel van Diepen^1^

^1^Department of Clinical Epidemiology, Leiden University Medical Center, Leiden, The Netherlands

^2^Parnassia Groep, The Hague, The Netherlands

^3^Department of Internal Medicine, Leiden University Medical Center, Leiden, The Netherlands

^4^Department of Internal Medicine, St. Antonius Hospital, Nieuwegein, The Netherlands

^5^Dutch Kidney Patients Association, Bussum, The Netherlands

**Corresponding author:**Jet Milders

[J.Milders@lumc.nl](mailto:J.Milders@lumc.nl)

Tel: +31623547842
Department of Clinical Epidemiology, Leiden University Medical Center, 2 Albinusdreef,

Leiden 2300RC, The Netherlands.**Table S1. Checklist for Reporting Of Survey Studies (CROSS) (1)**

| **Section/topic** | **Item** | **Item description** | **Reported on page #** |
| --- | --- | --- | --- |
| **Title and abstract** | | |  |
| Title and abstract | 1a | State the word “survey” along with a commonly used term in title or abstract to introduce the study’s design. | 1 |
|  | 1b | Provide an informative summary in the abstract, covering background, objectives, methods, findings/results, interpretation/discussion, and conclusions. | 2-3 |
| **Introduction** | | |  |
| Background | 2 | Provide a background about the rationale of study, what has been previously done, and why this survey is needed. | 4-5 |
| Purpose/aim | 3 | Identify specific purposes, aims, goals, or objectives of the study. | 5 |
| **Methods** | | |  |
| Study design | 4 | Specify the study design in the methods section with a commonly used term (e.g., cross-sectional or longitudinal). | 7 |
|  | 5a | Describe the questionnaire (e.g., number of sections, number of questions, number and names of instruments used). | 6-7, supplement |
| Data collection methods | 5b | Describe all questionnaire instruments that were used in the survey to measure particular concepts. Report target population, reported validity and reliability information, scoring/classification procedure, and reference links (if any). | 6-8 |
|  | 5c | Provide information on pretesting of the questionnaire, if performed (in the article or in an online supplement). Report the method of pretesting, number of times questionnaire was pre-tested, number and demographics of participants used for pretesting, and the level of similarity of demographics between pre-testing participants and sample population. | 6-7, supplement |
|  | 5d | Questionnaire if possible, should be fully provided (in the article, or as appendices or as an online supplement). | supplement |
| Sample characteristics | 6a | Describe the study population (i.e., background, locations, eligibility criteria for participant inclusion in survey, exclusion criteria). | 7 |
|  | 6b | Describe the sampling techniques used (e.g., single stage or multistage sampling, simple random sampling, stratified sampling, cluster sampling, convenience sampling). Specify the locations of sample participants whenever clustered sampling was applied. | 7 |
|  | 6c | Provide information on sample size, along with details of sample size calculation. | 7, supplement |
|  | 6d | Describe how representative the sample is of the study population (or target population if possible), particularly for population-based surveys. | 7, 13 |
| Survey  administration | 7a | Provide information on modes of questionnaire administration, including the type and number of contacts, the location where the survey was conducted (e.g., outpatient room or by use of online tools, such as SurveyMonkey). | 7-8 |
|  | 7b | Provide information of survey’s time frame, such as periods of recruitment, exposure, and follow-up days. | 7 |
|  | 7c | Provide information on the entry process:  –>For non-web-based surveys, provide approaches to minimize human error in data entry.  –>For web-based surveys, provide approaches to prevent “multiple participation” of participants. | 7-8 |
| Study preparation | 8 | Describe any preparation process before conducting the survey (e.g., interviewers’ training process, advertising the survey). | 6-7 |
| Ethical considerations | 9a | Provide information on ethical approval for the survey if obtained, including informed consent, institutional review board [IRB] approval, Helsinki declaration, and good clinical practice [GCP] declaration (as appropriate). | 6 |
|  | 9b | Provide information about survey anonymity and confidentiality and describe what mechanisms were used to protect unauthorized access. | 7 |
| Statistical  analysis | 10a | Describe statistical methods and analytical approach. Report the statistical software that was used for data analysis. | 7-8 |
|  | 10b | Report any modification of variables used in the analysis, along with reference (if available). | N.A. |
|  | 10c | Report details about how missing data was handled. Include rate of missing items, missing data mechanism (i.e., missing completely at random [MCAR], missing at random [MAR] or missing not at random [MNAR]) and methods used to deal with missing data (e.g., multiple imputation). | 7-8 |
|  | 10d | State how non-response error was addressed. | N.A. |
|  | 10e | For longitudinal surveys, state how loss to follow-up was addressed. | N.A. |
|  | 10f | Indicate whether any methods such as weighting of items or propensity scores have been used to adjust for non-representativeness of the sample. | N.A. |
|  | 10g | Describe any sensitivity analysis conducted. | 8 |
| **Results** | | |  |
| Respondent characteristics | 11a | Report numbers of individuals at each stage of the study. Consider using a flow diagram, if possible. | 9 |
|  | 11b | Provide reasons for non-participation at each stage, if possible. | N.A. |
|  | 11c | Report response rate, present the definition of response rate or the formula used to calculate response rate. | 9 |
|  | 11d | Provide information to define how unique visitors are determined. Report number of unique visitors along with relevant proportions (e.g., view proportion, participation proportion, completion proportion). | 9 |
| Descriptive  results | 12 | Provide characteristics of study participants, as well as information on potential confounders and assessed outcomes. | 9,  table 1 |
| Main findings | 13a | Give unadjusted estimates and, if applicable, confounder-adjusted estimates along with 95% confidence intervals and p-values. | 9-12, supplement |
|  | 13b | For multivariable analysis, provide information on the model building process, model fit statistics, and model assumptions (as appropriate). | N.A. |
|  | 13c | Provide details about any sensitivity analysis performed. If there are considerable amount of missing data, report sensitivity analyses comparing the results of complete cases with that of the imputed dataset (if possible). | 12 |
| **Discussion** | | |  |
| Limitations | 14 | Discuss the limitations of the study, considering sources of potential biases and imprecisions, such as non-representativeness of sample, study design, important uncontrolled confounders. | 13 |
| Interpretations | 15 | Give a cautious overall interpretation of results, based on potential biases and imprecisions and suggest areas for future research. | 13-17 |
| Generalizability | 16 | Discuss the external validity of the results. | 13 |
| **Other sections** | | |  |
| Role of funding source | 17 | State whether any funding organization has had any roles in the survey’s design, implementation, and analysis. | 18 |
| Conflict of interest | 18 | Declare any potential conflict of interest. | 18 |
| Acknowledgements | 19 | Provide names of organizations/persons that are acknowledged along with their contribution to the research. | 18 |

**DETAILED METHODS**

**Explanation of the two-phase pilot**

The survey was tested during a two-phase pilot: First, three volunteers of the Dutch Kidney Patients Association were invited to share their thoughts on the survey during an online video meeting with two of the authors (JM and CLR). Topics discussed were, amongst others, their general impression of the survey, the included questions, the amount of time it took them to complete the survey and the experienced burden of filling in the survey. Their feedback was incorporated into the first version of the survey. Second, the survey and the accompanying informational letter were sent to two volunteers of the Dutch Kidney Patients Association. The aims of this second phase were to test whether the data gathering in Castor EDC worked well, and to test the overall process of spreading the survey. Furthermore, participants of this second phase also had the opportunity to provide us with any additional feedback during an online video meeting. Again, their feedback was incorporated and the survey was finalized.

**Explanation of the populations (CKD, dialysis and kidney transplantation)**

The CKD population was defined as patients that were not undergoing KRT (dialysis or kidney transplantation). The dialysis group consists of patients that were undergoing either hemodialysis or peritoneal dialysis at the time of completing in the survey. Patients that had undergone a kidney transplantation and that were not undergoing dialysis at the time were categorized as kidney transplantation patients for the analyses.

**The survey**

The final survey consisted of three main parts: 1) demographics, 2) considerations about the future, and 3) prognostic information. In the first part, participants were asked to provide general information, such as their age, gender, educational level, living situation and treatment type. In the second part, participants were asked if they ever think about their future with CKD and if they discuss this with their nephrologist. Finally, in the last part of the survey, a variety of visual analogue scale (VAS) (0-100, not at all-very much), multiple-choice and open-ended questions were asked to assess to which extent participants wanted to know more about their future with CKD and which specific topics they were interested in regarding their prognosis.

**FIGURES AND TABLES**

**Figure S1.**


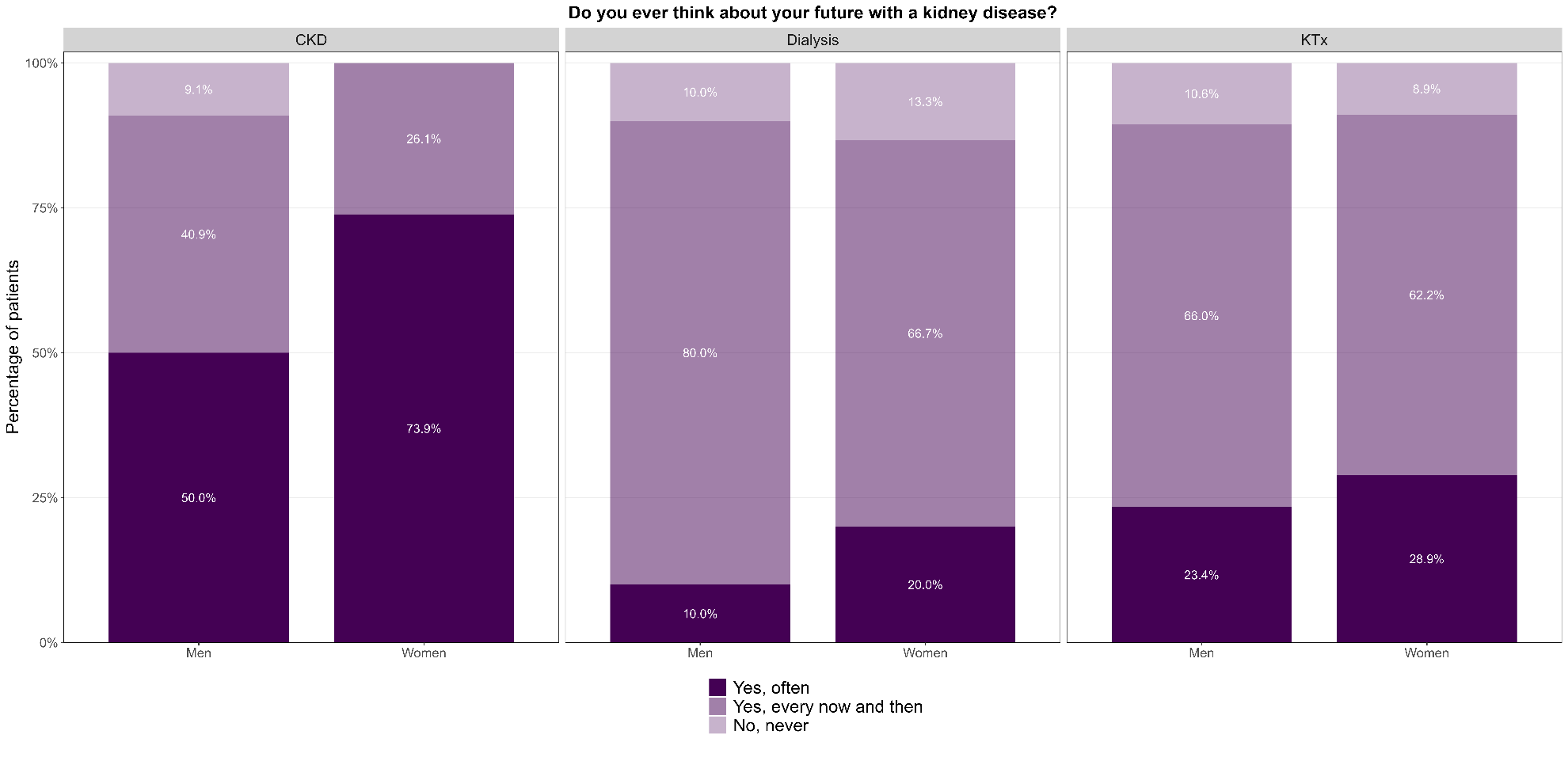

One person filled in “Other” for gender. This person answered “Yes, often”.

**Figure S2.
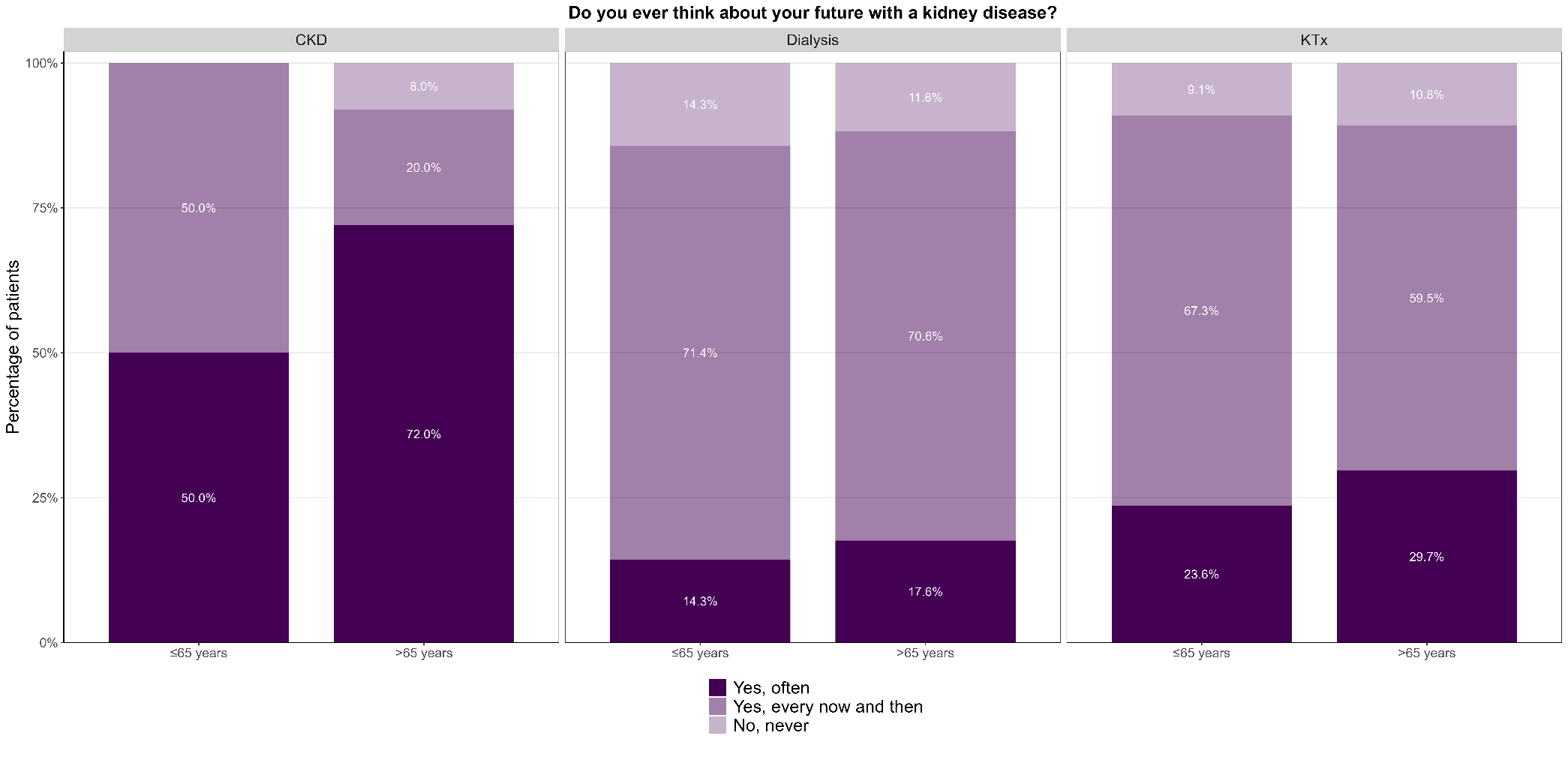
**

**Figure S3.**


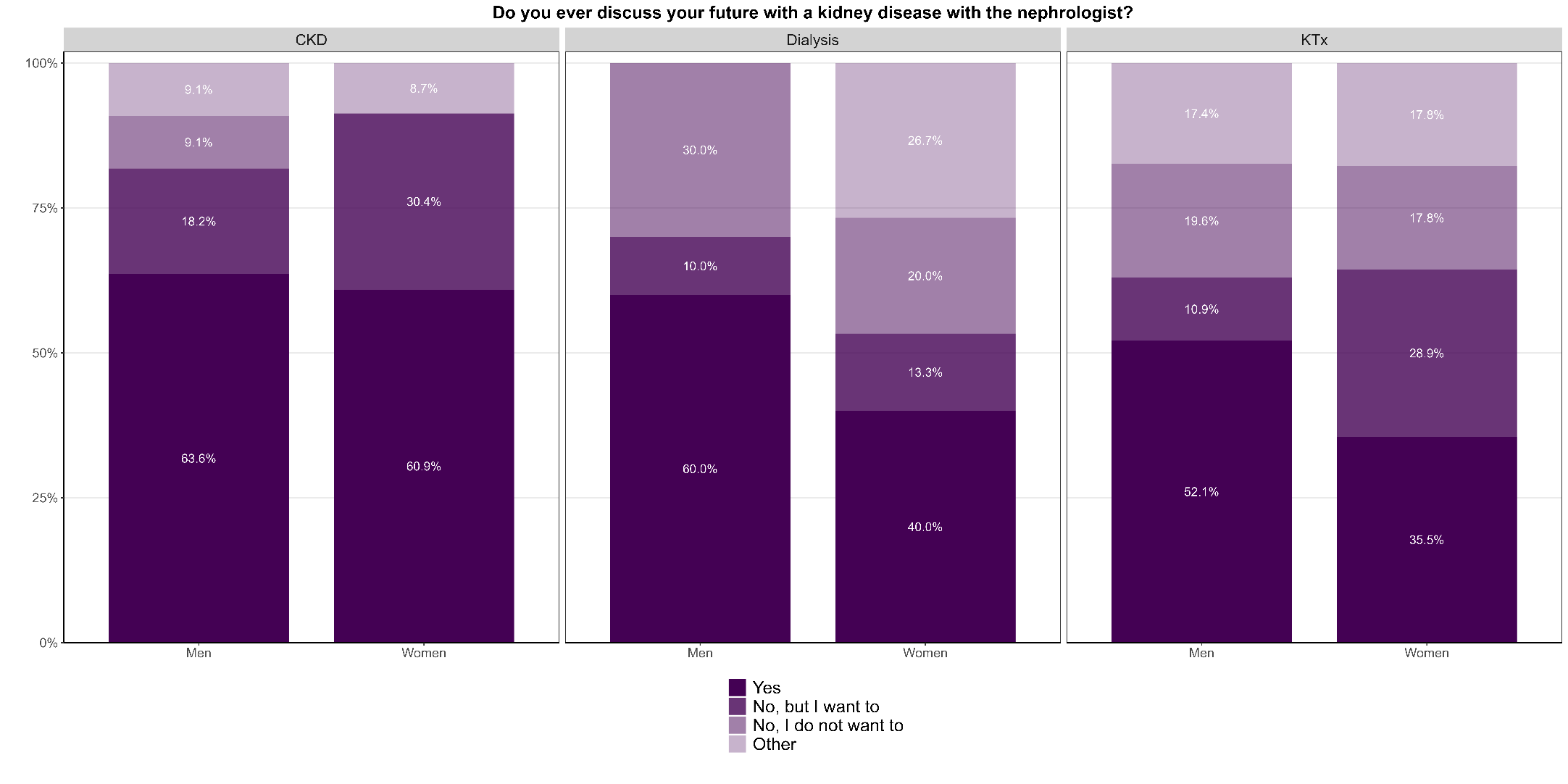

One person filled in “Other” for gender. This person answered “Yes” to question B.

**Figure S4.
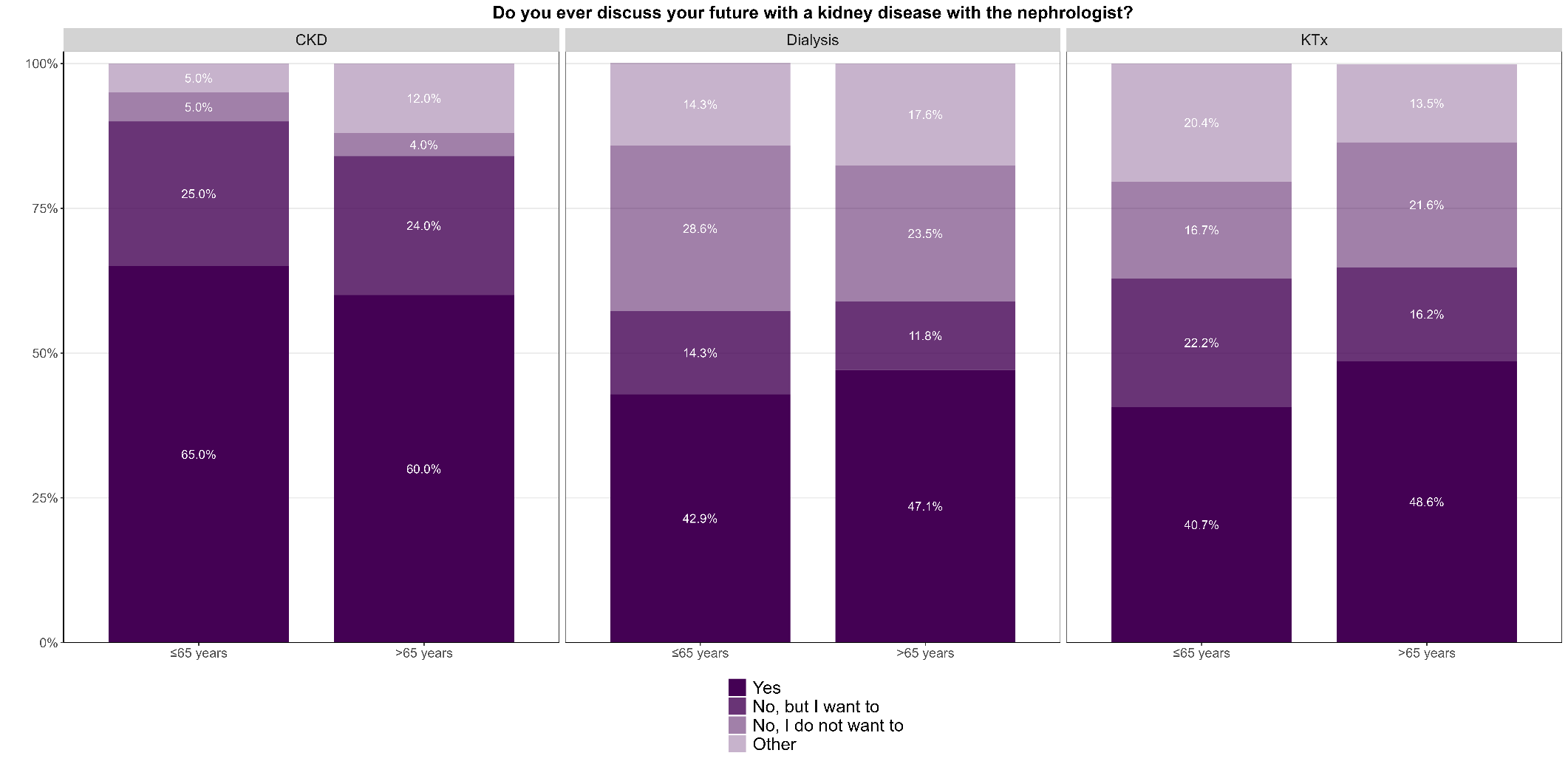
**

**Figure S5.**


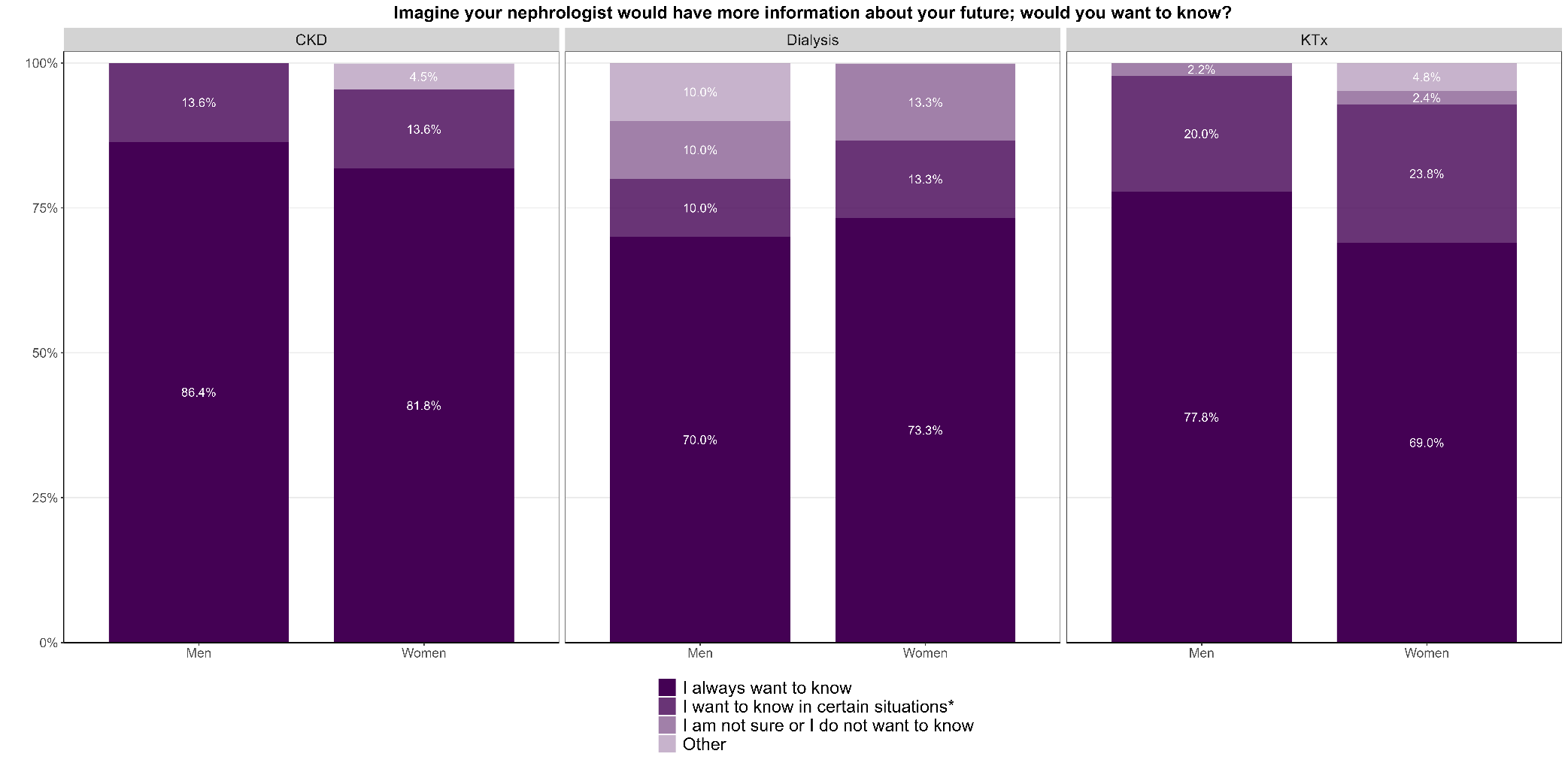

*Certain situations consist of the following: only in case of good news, if we can do something to prevent it and if it supports me in making a treatment decision.
One person filled in “Other” for gender. This person answered “I always want to know” to question C.

**Figure S6.**


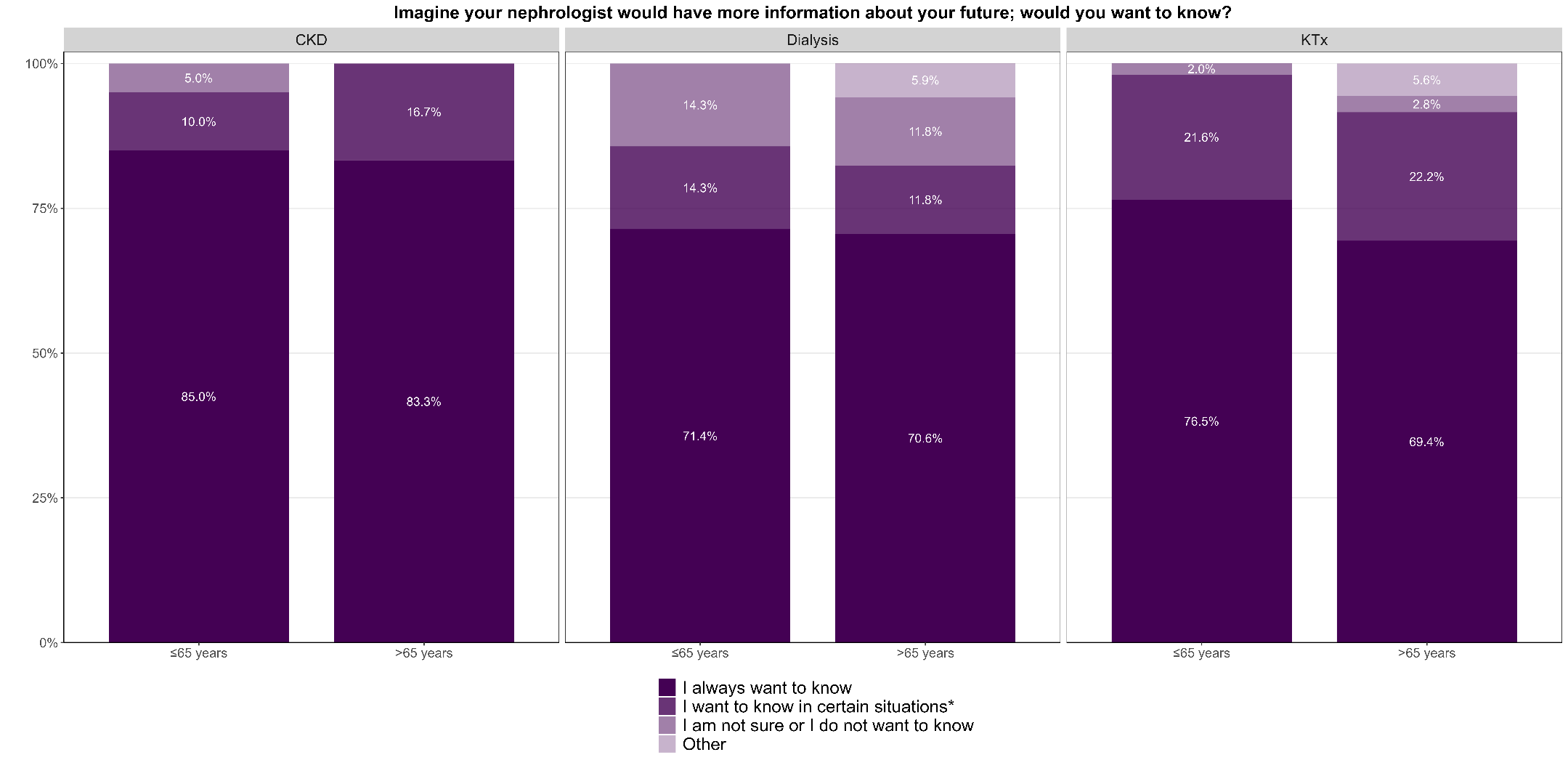

*Certain situations consist of the following: only in case of good news, if we can do something to prevent it and if it supports me in making a treatment decision.

|  | **Gender** | |
| --- | --- | --- |
|  | **Men (n = 79)** | **Women (n = 83)** |
| Health complaints | 69.9 (28.1) | 65.7 (30.6) |
| Disease progression and comorbidities | 55.9 (33.3) | 56.7 (35.0) |
| Laboratory values and measurements | 75.4 (30.2) | 80.0 (29.3) |
| Dialysis | 47.5 (36.0) | 46.9 (36.9) |
| Kidney transplantation | 52.3 (40.6) | 52.1 (40.3) |
| Conservative management | 37.3 (34.6) | 40.1 (35.0) |
| Physical well-being | 65.9 (34.2) | 69.0 (34.7) |
| Mental well-being | 61.5 (37.2) | 61.0 (35.9) |
| Social participation | 56.2 (37.1) | 59.2 (37.6) |

**Table S2. Mean (SD) rating per outcome category stratified by gender**

One person filled in “Other” for gender.

|  | **Age** | |
| --- | --- | --- |
|  | **≤65 years (n = 82)** | **>65 years (n = 79)** |
| Health complaints | 68.9 (26.2) | 66.3 (32.6) |
| Disease progression and comorbidities | 59.6 (29.2) | 52.4 (38.4) |
| Laboratory values and measurements | 78.8 (27.9) | 76.3 (31.8) |
| Dialysis | 49.8 (36.0) | 44.0 (36.6) |
| Kidney transplantation | 63.3 (37.8) | 40.3 (39.6) |
| Conservative management | 40.2 (33.7) | 37.7 (35.8) |
| Physical well-being | 72.0 (32.4) | 62.1 (35.9) |
| Mental well-being | 62.5 (34.4) | 59.5 (38.8) |
| Social participation | 62.0 (35.9) | 53.8 (38.3) |

**Table S3. Mean (SD) rating per outcome category stratified by age**

**Figure S7.**

**
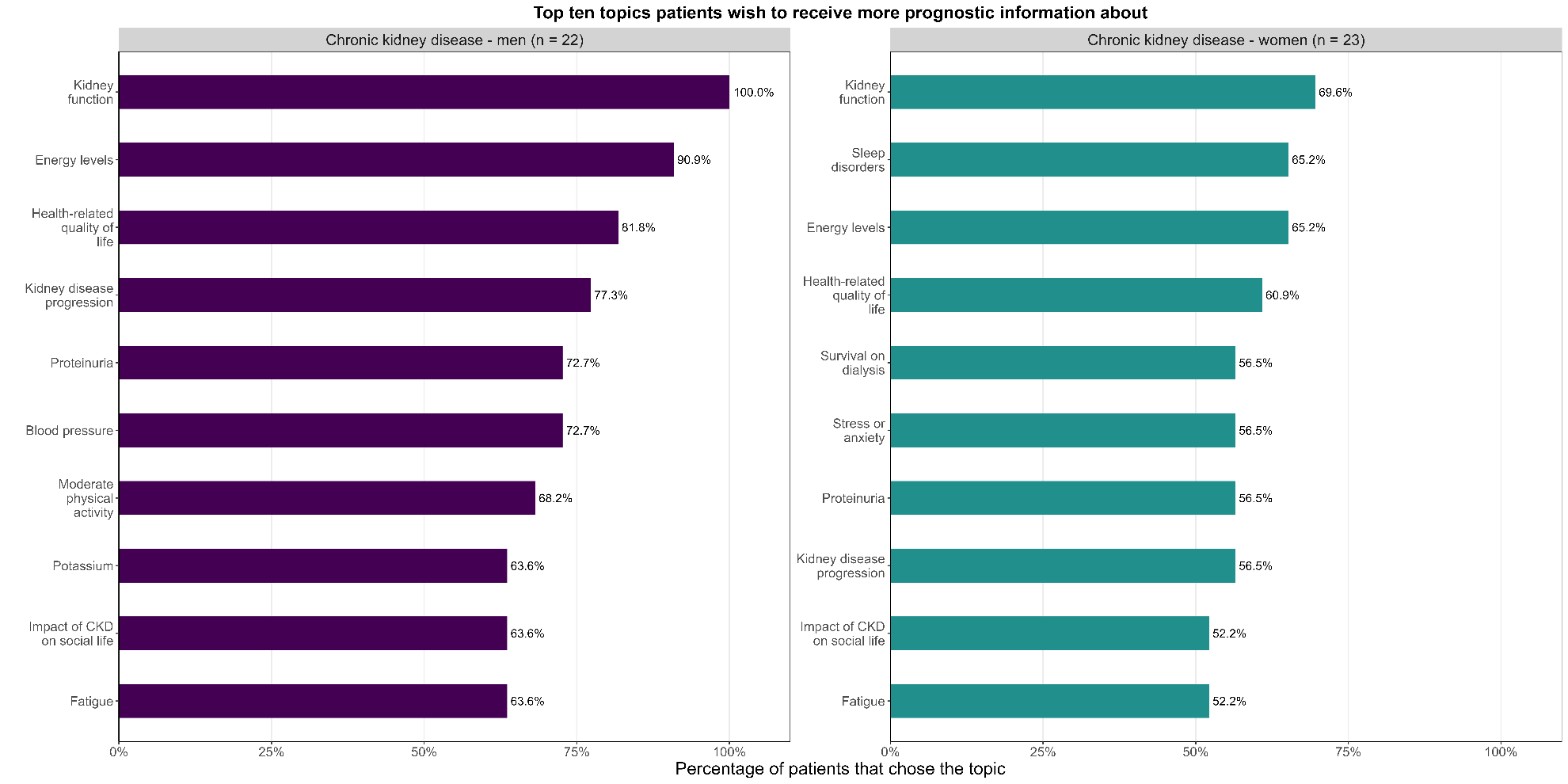
**

For men, hemoglobin was also chosen by 63.6% of patients. For women, blood pressure, potassium and hemoglobin were also chosen by 52.2% of patients.

**Figure S8.
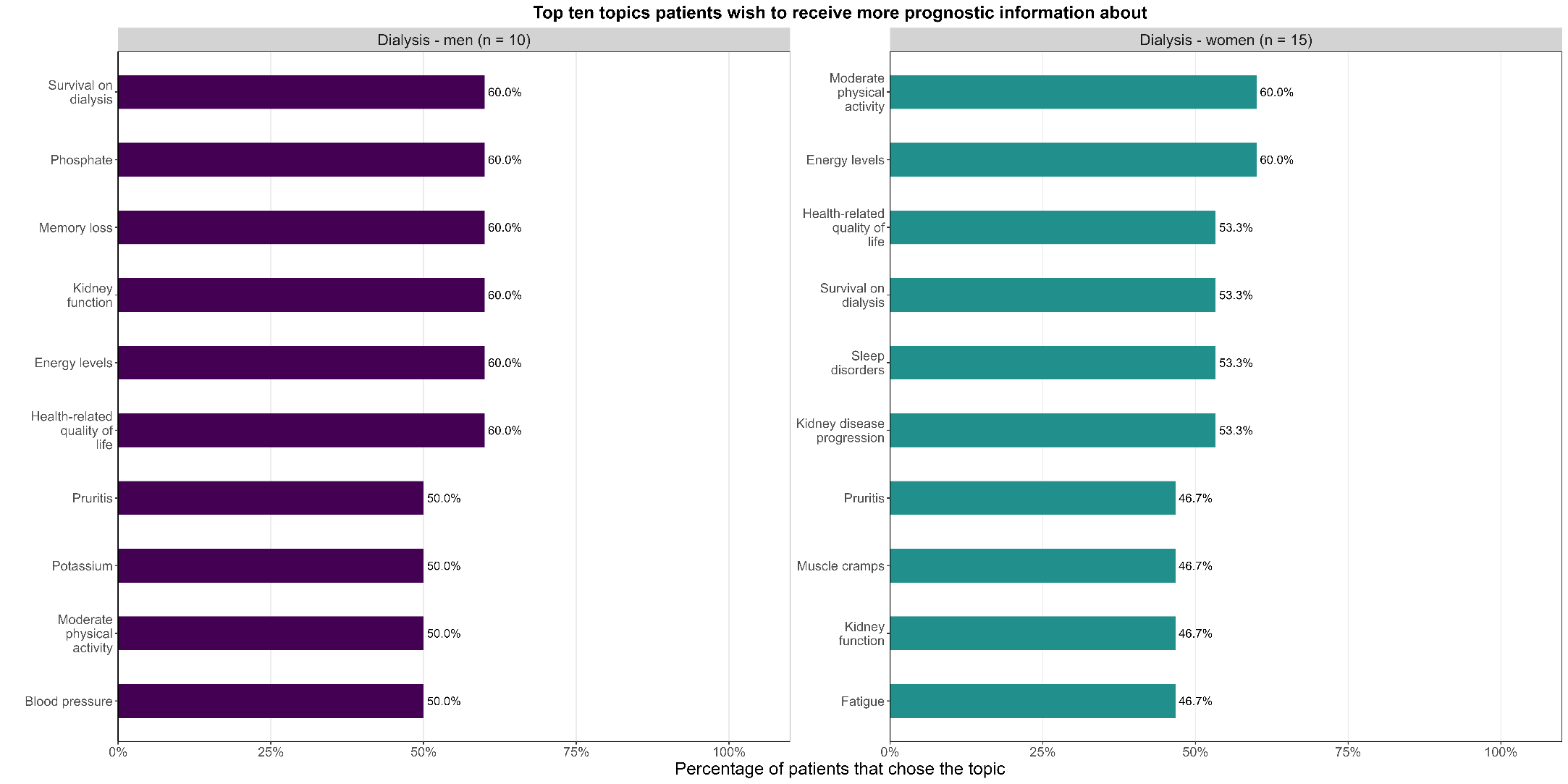
**For women, cardiovascular disease, potassium, concentration problems, calcium, hemoglobin and phosphate were also chosen by 46.7% of patients.

**Figure S9.
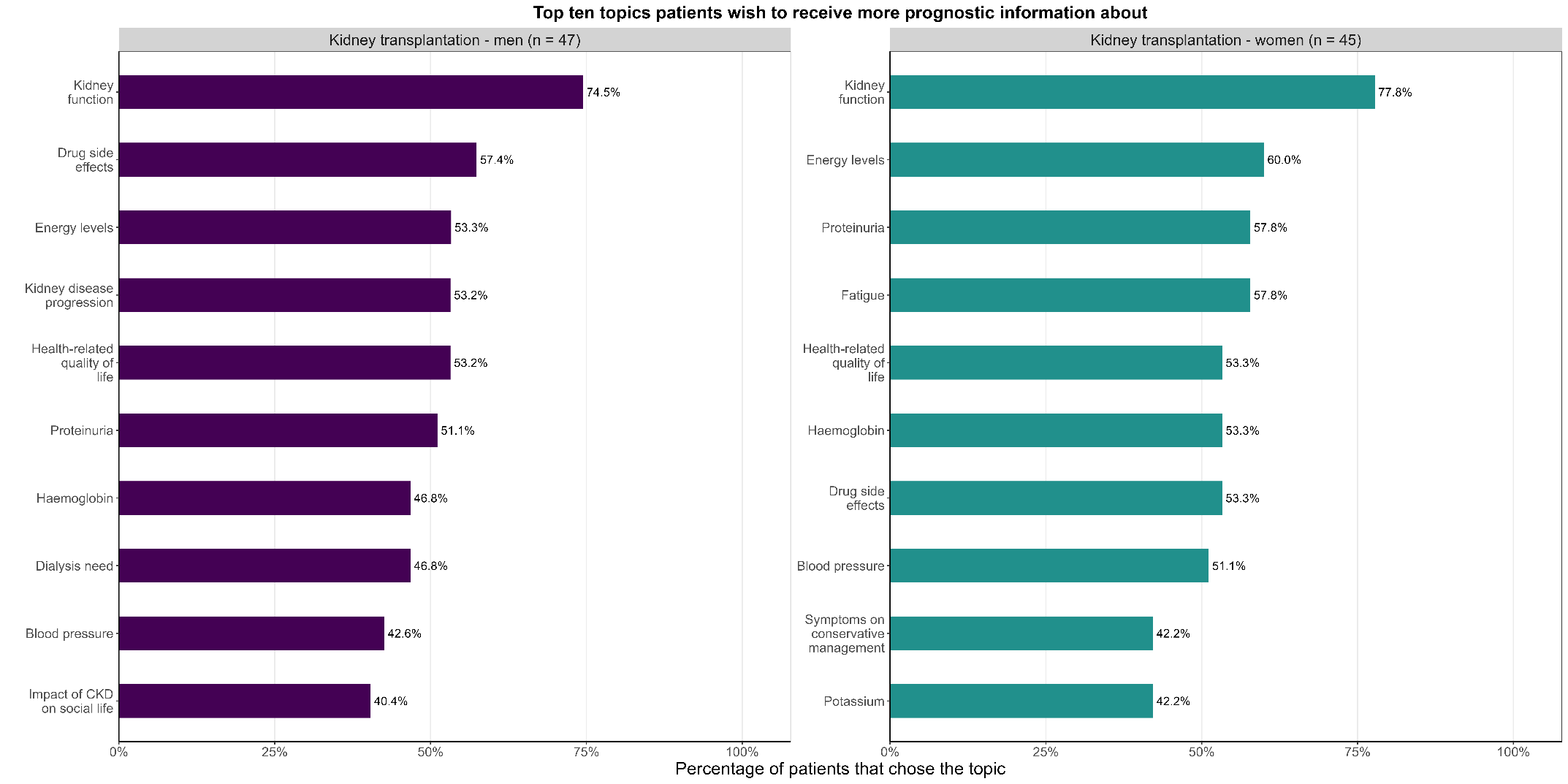
**

**Figure S10.**

**
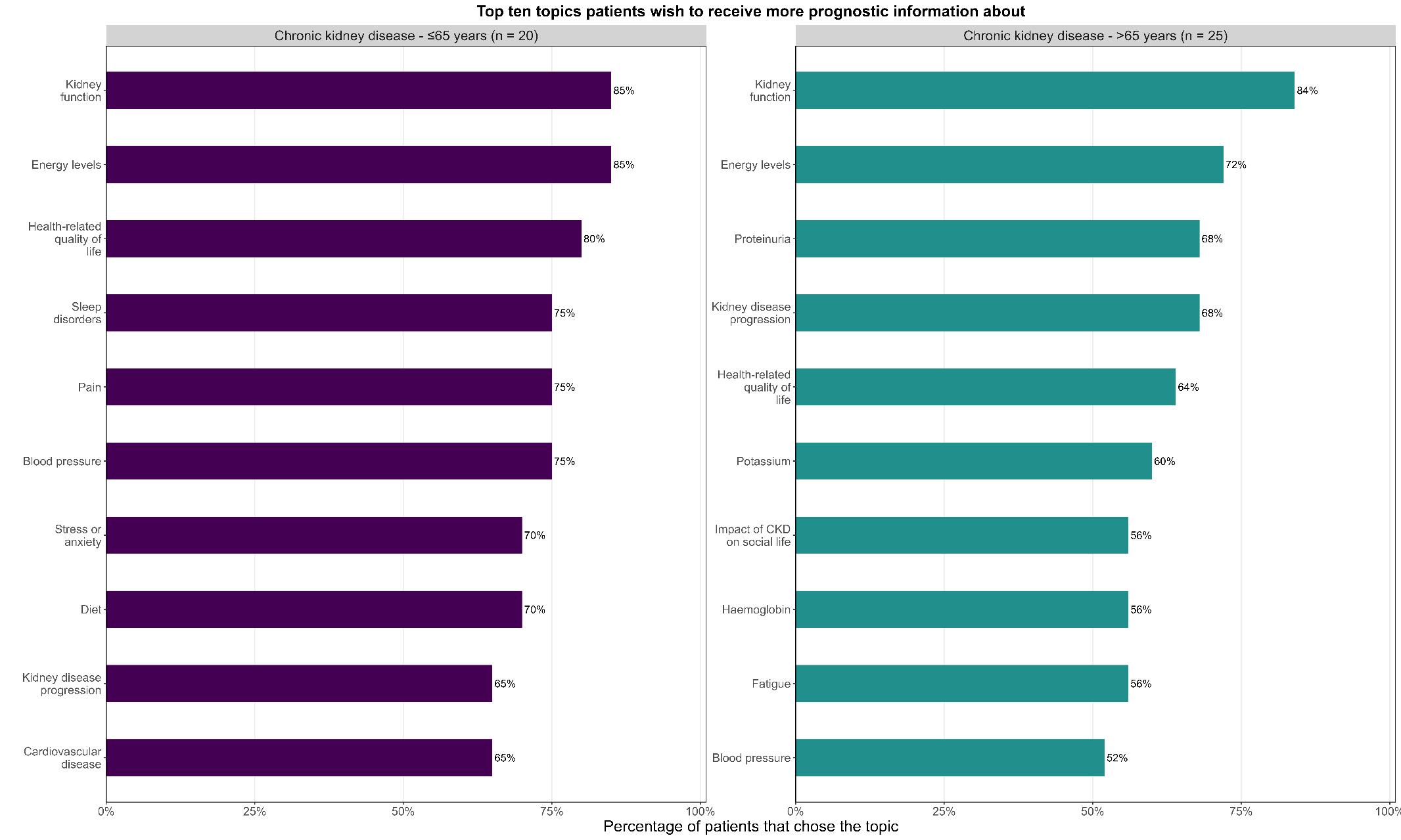
**For patients ≤65 years, moderate physical activity was also chosen by 65% of patients.

**Figure S11.**

**
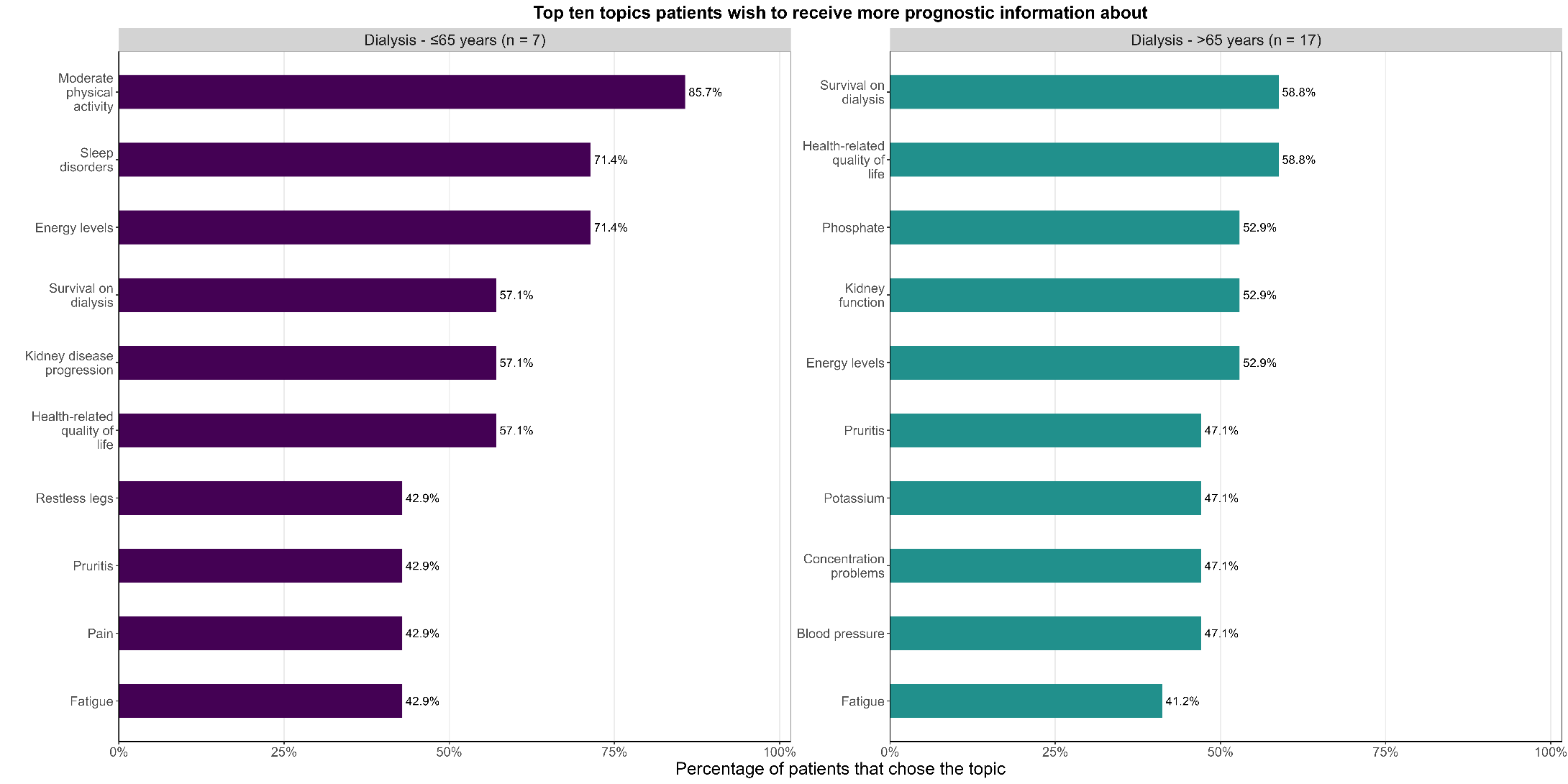
**For patients ≤65 years, the following were also chosen by 42.9% of patients: Muscle cramps, dry skin, gastrointestinal issues, life expectancy with kidney disease, kidney function, proteinuria, potassium, phosphate, calcium, hemoglobin, dialysis efficacy, surgical complications, activities of daily living, ability to work.
Fort patients >65 years the following were also chosen by 41.2% of patients: muscle cramps, cardiovascular disease, life expectancy, calcium, hemoglobin and moderate physical activity.

**Figure S12.**

**
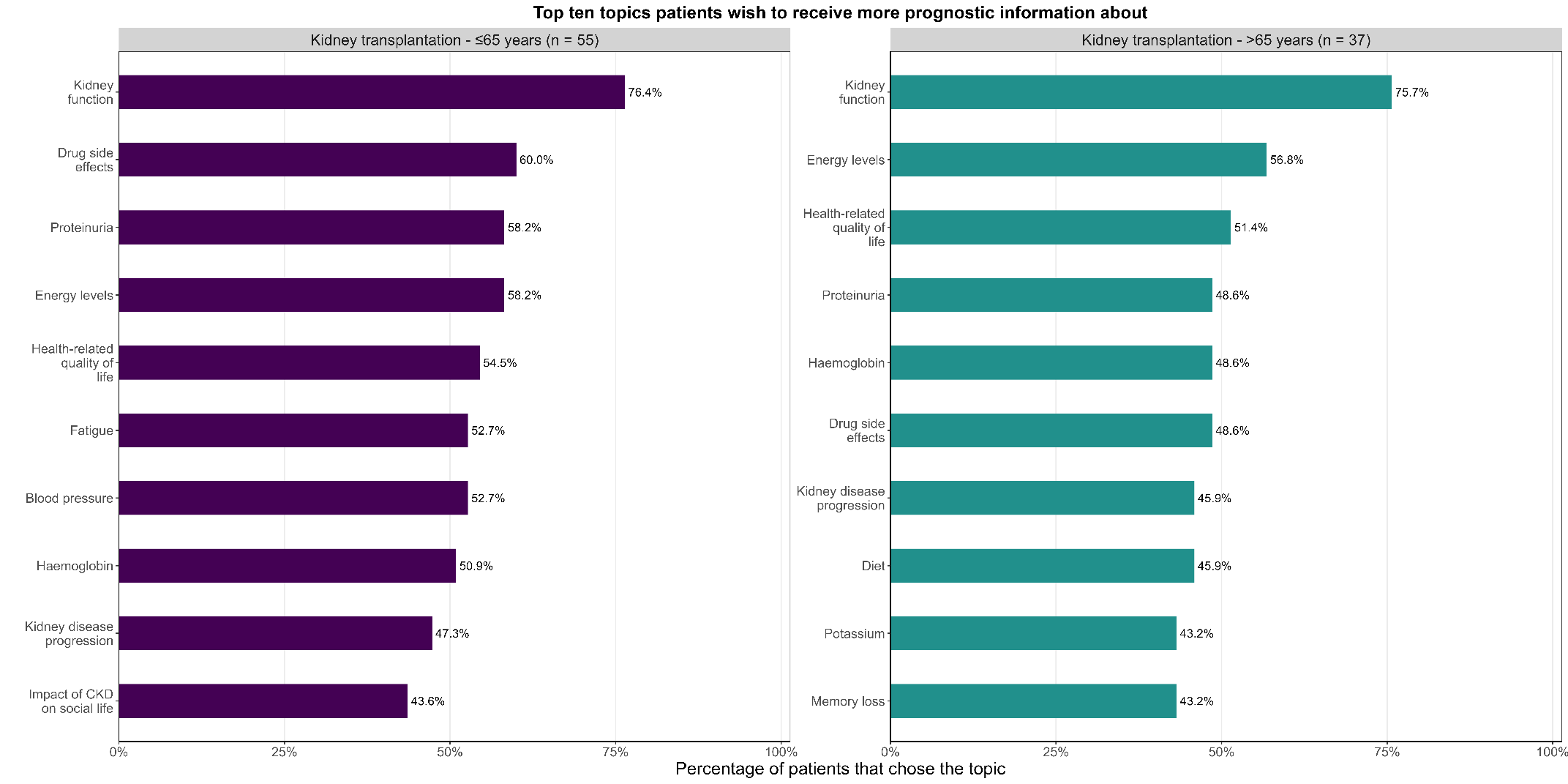
**

For patients ≤65 years cardiovascular disease was also chosen by 43.6% of patients.

**Figure S13.**

**
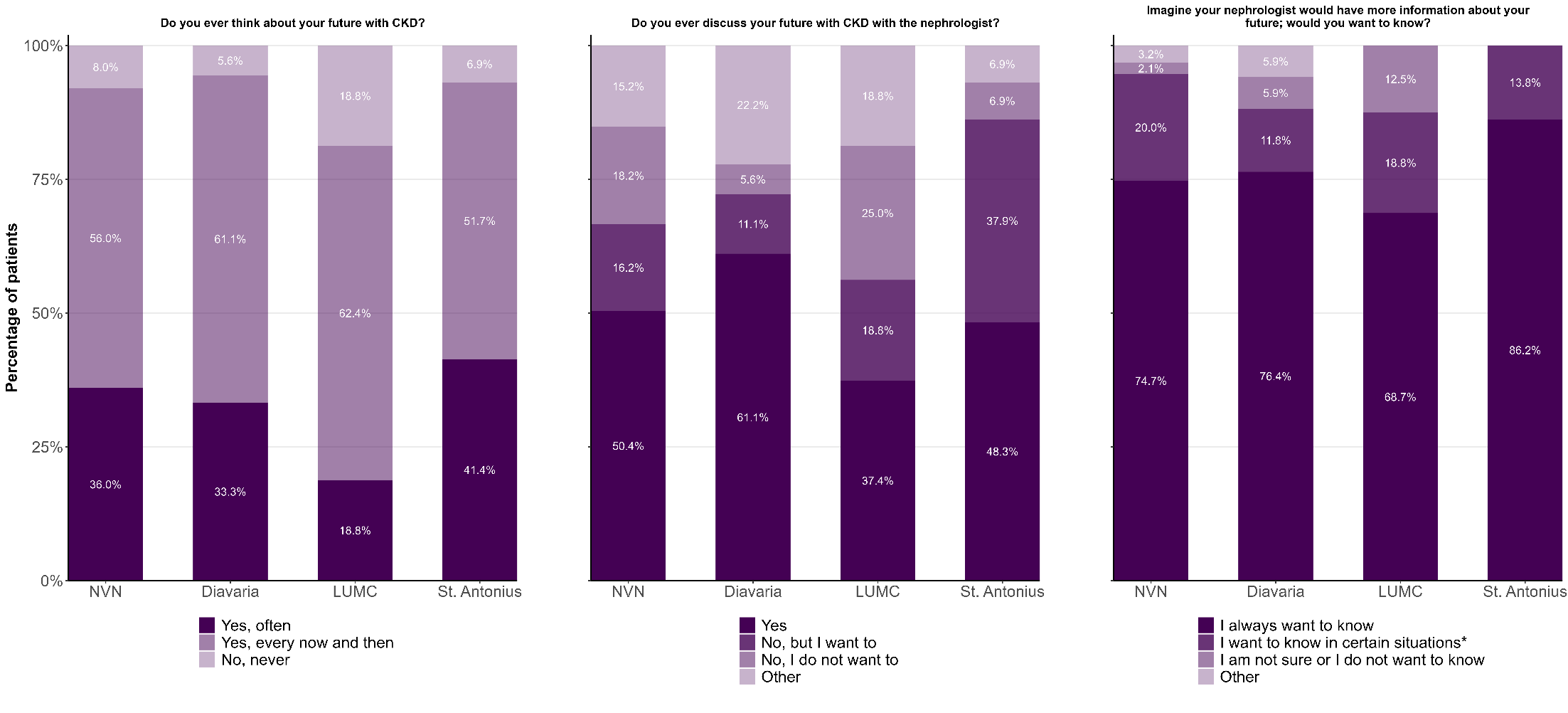
**

CKD = chronic kidney disease, KTx = kidney transplantation
*Certain situations consist of the following answer options: only in case of good news, if we can do something to prevent it and if it supports me in making a treatment decision.

**REFERENCES**

1. Sharma A, Minh Duc NT, Luu Lam Thang T, Nam NH, Ng SJ, Abbas KS, et al. A Consensus-Based Checklist for Reporting of Survey Studies (CROSS). J Gen Intern Med. 2021;36(10):3179-87.
